# A Series of 14 Polish Patients with Thrombotic Events and PC Deficiency-Novel c.401-1G>A *PROC* Gene Splice Site Mutation in a Patient with Aneurysms

**DOI:** 10.3390/genes13050733

**Published:** 2022-04-22

**Authors:** Anna Weronska, Daniel P. Potaczek, Julia Oto, Pilar Medina, Anetta Undas, Ewa Wypasek

**Affiliations:** 1 Faculty of Medicine and Health Sciences, Andrzej Frycz Modrzewski Krakow University, 30-705 Krakow, Poland; anna.weronska@neuroplay.pl; 2 Translational Inflammation Research Division & Core Facility for Single Cell Multiomics, Medical Faculty, Biochemical Pharmacological Center (BPC), Philipps University of Marburg, 35043 Marburg, Germany; potaczek@staff.uni-marburg.de; 3 Haemostasis, Thrombosis, Arteriosclerosis and Vascular Biology Research Group, Medical Research Institute Hospital La Fe, 46026 Valencia, Spain; juliaotomartinez@gmail.com (J.O.); medina_pil@gva.es (P.M.); 4 Institute of Cardiology, Jagiellonian University School of Medicine, 31-202 Krakow, Poland; mmundas@cyf-kr.edu.pl; 5 John Paul II Hospital, 80 Prądnicka Street, 31-202 Krakow, Poland

**Keywords:** protein C deficiency, gene mutations, venous thromboembolism, family study

## Abstract

Objectives: Protein C (PC) deficiency is an inherited thrombophilia with a prevalence of 0.5% in the general population and 3% in subjects with a first-time deep vein thrombosis (DVT). Here we report a series of 14 PC-deficient Polish patients with comprehensive clinical and molecular characteristics, including long-term follow-up data and a deep mutational analysis of the *PROC* gene. Patients and Methods: Fourteen unrelated probands (mean ± SD age 43.8 ± 13.0 years) with suspicion of PC deficiency, who experienced thromboembolic events and a majority of whom received anticoagulants (92.8%), were screened for *PROC* mutations by sequencing the nine *PROC* exons and their flanking intron regions. Results: Ten probands (71.4%) had missense mutations, two patients (14.3%) carried nonsense variants, and the other two subjects (14.3%) had splice-site mutations, the latter including the c.401-1G>A variant, reported here for the very first time. The proband carrying the c.401-1A allele had a hepatic artery aneurysm with a highly positive family history of aneurysms and the absence of any mutations known to predispose to this vascular anomaly. Conclusion: A novel detrimental *PROC* mutation was identified in a family with aneurysms, which might suggest yet unclear links of thrombophilia to vascular anomalies, including aneurysms at atypical locations in women. The present case series also supports data indicating that novel oral anticoagulants (NOACs) are effective in PC deficient patients.

## 1. Introduction

Protein C (PC) is a vitamin K-dependent serine protease that is activated at the endothelial surface and, in the presence of protein S (PS), phospholipid and calcium inhibits thrombin generation by proteolytic inactivation of factors Va (FVa) and VIIIa [1]. Besides its well-recognized anticoagulant properties, the PC system exerts anti-apoptotic and anti-inflammatory effects [2].

The prevalence of PC deficiency is estimated at 0.5% in the general population and 3% in patients with a first-time deep vein thrombosis (DVT) [2,3]. The clinical presentation of PC deficiency is highly variable ranging from asymptomatic individuals, through venous thromboembolism (VTE), to acute life-threatening complications like purpura fulminans, which is likely related to the degree of deficiency as well as the presence of other acquired or inherited thrombophilic factors [4].

Arterial thrombosis occurs uncommonly among PC-deficient subjects, but increased rates of myocardial infarction (MI) and ischemic stroke have been reported in case reports and a large cohort study [5,6,7]. Furthermore, young survivors of MI have low levels of activated protein C, which inversely correlates with the severity of coronary lesions [8].

Most patients with PC deficiency are heterozygous carriers of *PROC* mutations. According to the Human Gene Mutation Database (HGMD, http://www.hgmd.cf.ac.uk/ac/index.php accessed on 1 March 2022), 509 *PROC* mutations have been reported as causes of PC deficiency, with a predominance of missense/nonsense mutations (74%) followed by splicing-affecting variants and small deletions (8% each).

We were the first to present cases with genetically confirmed PC-deficiency in the Polish population [9,10]. Here we report a series of 14 PC-deficient Polish patients with their comprehensive clinical and genetic characteristics such as long-term follow-up data and molecular identification of PC-deficiency underlying mutations, including a novel *PROC* splice site variant associated with this thrombophilia.

## 2. Materials and Methods

### 2.1. Patients

We evaluated fourteen consecutive unrelated patients (probands) with suspicion of PC-deficiency, who experienced thromboembolic events and were therefore referred to the Center for Coagulation Disorders at the John Paul II Hospital, Krakow, Poland between September 2017 and July 2021.

The diagnoses of VTE and ischemic stroke were established as previously described [9]. Briefly, the diagnosis of DVT was based on a positive finding of color duplex sonography (the visualization of an intraluminal thrombus in the calf, popliteal, femoral, or iliac vein) and the diagnosis of pulmonary embolism (PE) was established on the basis of the presence of typical symptoms and positive results of computed tomography (CT) pulmonary angiography. The data on the family history of thromboembolic events in the first and/or second-degree relatives of the probands were collected. Family history was regarded as positive if VTE was diagnosed in at least one first-degree relative. The risk factors i.e., pregnancy, oral contraceptives, known malignancy, immobilization, surgery, or trauma were also analyzed.

The patients were followed until December 2021 on a 6–12 month basis, including a visit at the center or telephone contact. The novel oral anticoagulants (NOACs) treatment continued throughout the follow-up period. New documented thrombotic events or bleedings were recorded, considering major or clinically relevant non-major bleeding (CRNMB) according to the International Society on Thrombosis and Haemostasis (ISTH) criteria [11]. The retrospective analysis was a part of a routine clinical diagnostic evaluation and, therefore, the approval of the Bioethical Commission was not required.

### 2.2. Blood Samples

Blood samples were collected from an antecubital vein into tubes containing citrate anticoagulant (9:1 of 0.106 M sodium citrate), centrifuged at 2500× *g* at room temperature for 20 min, and processed immediately or stored in aliquots at −80 °C until analysis. Whole blood samples for DNA isolation were drawn into EDTA-K3 collection tubes and stored in aliquots at −80 °C until processing.

### 2.3. Laboratory Tests

The tests were performed at least 3 months after the thromboembolic event and after temporary withdrawal of vitamin K antagonist (for at least 10 days) or direct oral anticoagulant (NOAC; for at least 24 h). The probands were screened for thrombophilia as described [9].

Plasma PC activity was measured in citrated plasma using a chromogenic assay (Berichrom Protein C, Siemens Healthcare Diagnostics, Erlangen, Germany; reference range 70–140%) and a clot-based assay (Protein C coag, Siemens Healthcare Diagnostics; reference range 70–140%). PC antigen was assessed in citrated plasma by an enzyme-linked immunosorbent assay (Asserachrom Protein C, Diagnostica Stago, Asnieres, France; reference range 70–140%). PC deficiency was classified as previously described [9], i.e., type I-quantitative deficiency with a decreased synthesis of abnormal protein, which results in decreased activity and plasma concentration of PC, and type II-qualitative deficiency (decline in PC activity).

### 2.4. Genetic Analysis

Genomic DNA was extracted from EDTA whole blood according to the manufacturer’s protocol, using Sherlock AX Purification Kit (A&A Biotechnology, Gdańsk, Poland) and stored at 4 °C until analysis. The nine exons and flanking regions of the *PROC* (NM_000312.3) gene were analyzed as previously described [12].

An adapter-tagged DNA library was purified, amplified, and enriched using SureSelect XT capture library (Agilent Technologies, Santa Clara, CA, USA). Sequencing of the libraries was performed on a NextSeq 550 instrument (Illumina, San Diego, CA, USA). Primers were designed so that pseudogene amplification was avoided.

For patients carrying the c.401-1G>A mutation, a genetic analysis of the coding exons and the flanking intron regions of *FBN1, TGFBR1*, *TGFBR2*, *TGFB2, SMAD2*, *SMAD3*, *ACTA2*, *COL3A1, MYH11*, *TGFB3*, and *SKI* was also performed. The generated amplicons were then analyzed by Illumina Sequencing By Synthesis (SBS) technology (MiSeq Personal Sequencer, Illumina, San Diego, CA, USA). All variants detected were confirmed by Sanger sequencing.

The functional interpretation of mutations potentially altering the splicing of *PROC* exons was evaluated using the following free software: Human Splicing Finder (https://www.genomnis.com/access-hsf accessed on 1 March 2022), NetGene2 (https://services.healthtech.dtu.dk/service.php?NetGene2-2.42 accessed on 1 March 2022), Splice Site Predictor (https://www.fruitfly.org/seq_tools/splice.html accessed on 1 March 2022) and ASSP (http://wangcomputing.com/assp/ accessed on 1 March 2022).

### 2.5. Statistical Analysis

Continuous variables are given as mean ± standard deviation. Statistical calculations were performed using STATISTICA Version 13.3 (StatSoft, Inc., Tulsa, OK, USA).

## 3. Results

As shown in Table 1, the final analysis included fourteen PC-deficient probands aged 43.8 ± 13.0 years (women, 64.3%), including 13 patients without any other thrombophilia and one patient with factor V Leiden mutation in heterozygosity (patient No. 9). The mean age of the first thromboembolic event was 41.8 ± 13.1 years. Positive family history was found in seven (50%) patients.

The mean PC activity assessed by chromogenic assay was 58.4 ± 9.8% and by clot-based assay, was 49.6 ± 9.8%, while the mean total PC was 59.1 ± 19.6%. Eight patients (57.1%) had a type I PC deficiency and four individuals (28.6%) had a type II PC deficiency. In two patients (14.3%), the type of PC deficiency could not be ascertained due to incomplete laboratory data.

Most of the probands experienced isolated DVT (35.7%), followed by DVT with concomitant pulmonary embolism (PE; 21.4%). As few as three (21.4%) events were unprovoked. Ischemic stroke occurred in a single patient, however, a relationship between arterial thromboembolic events and natural anticoagulant deficiency has not been established in Polish patients [9].

Interestingly enough, a hepatic artery aneurysm occurred in a 56-year-old woman carrying a novel *PROC* mutation identified herein (c.401-1G>A). In addition, one DVT complicated by PE occurred in a 63-year-old proband carrying this *PROC* mutation, so far not described in the Polish population, but found in Hungarian patients with PC deficiency and predicted by Speker et al. (c.759C>A; p.His253Gln) [13] to be pathogenic. In this patient with comorbidities such as hypertension, obesity, hypercholesterolemia, and hyperglycemia, a dilated ascending aorta was found after the VTE event.

Ten probands (71.4%) received NOACs; three patients (21.4%) received dabigatran (150 mg bid), three apixaban (2.5 mg bid or 5 mg bid) and four (28.6%) rivaroxaban (15 mg or 20 mg qd). Among patients in whom oral anticoagulants had not been administered, one patient was on clopidogrel (75 mg/day), one on sulodexide (250 SLU bid), and one on acetylsalicylic acid (ASA; 75 mg). The first of those, had hyperlipidemia, coronary artery disease, and prior ischemic stroke over ten years ago, and initially did not receive any prophylaxis due to earlier Quincke’s edema after receiving a high dose of ASA. Five years ago, despite restarting ASA, the patient experienced another ischemic stroke, and therefore, clopidogrel (75 mg/day) was administered. One patient did not receive anticoagulant agents due to the long time span since the VTE event and the absence of thrombotic manifestation.

During a median follow-up of 31 months (IQR, 13–54), neither thrombotic nor bleeding events were observed.

### 3.1. PROC Mutations

Ten probands (71.4%) had missense *PROC* mutations, two patients (14.3%) carried splice-site variants, and in two individuals (14.3%) nonsense mutations were detected (Table 1). In all of the cases, the status of the variants identified in the patients was heterozygous. The p.His253Gln (c.759C>A) variant was identified in five unrelated patients, while the p.Cys106Arg (c.316T>C) in two unrelated individuals. To the very best of our knowledge, the c.401-1G>A splice site mutation (identified in patient No. 1) is reported here for the first time. The in silico analysis showed that the presence of the mutation disrupted the acceptor splicing site of intron 5. Consequently, the splicing rate could be altered, and/or a fragment of intron 5 might be incorporated into the final protein product.

### 3.2. Novel PROC Gene Splice Site Mutation and Aneurysms

**Figure 1:**
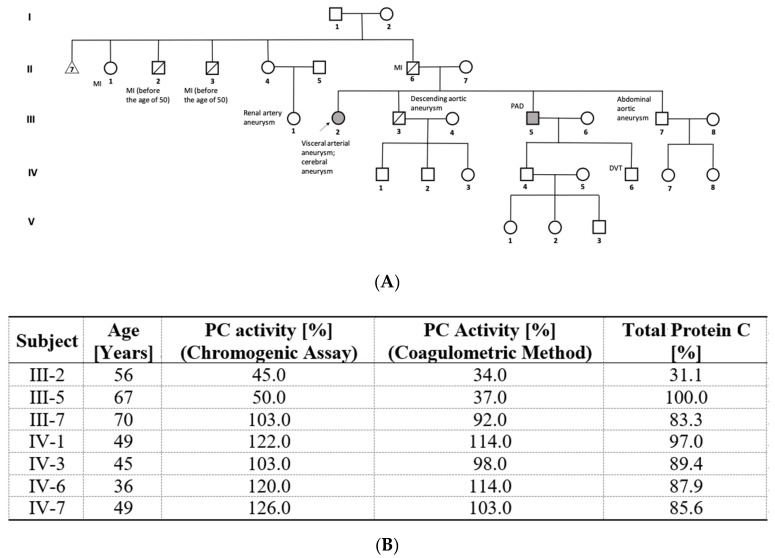
(**A**): pedigree of patient with novel c.401-1G>A *PROC* gene splice site mutation. The mutation carriers marked with grey and the proband indicated with an arrow. DVT denotes deep vein thrombosis; PAD, peripheral arterial disease; MI, myocardial infarction. (**B**): demographic and laboratory data for the subjects available for genetic and biochemical analyses. The reference ranges: PC activity, 70–140%; total protein C, 60–140%.

A 56-year-old Polish female patient with a hepatic artery aneurysm and a highly positive family history of aneurysms was referred to our Centre to be tested for thrombophilia, in whom we identified a novel c.401-1G>A *PROC* gene splice site mutation. Figure 1 shows the pedigree of the proband (III-2). The proband’s first brother (III-7) is a 70-year-old man with PC levels of 103% and an abdominal aortic aneurysm. Herein, we observe the absence of segregation between the variant and the deficiency. The second brother (III-3) died of dilation of descending aorta aneurysm without previous surgical management, but PC levels were not assessed in this patient. The third brother (III-5) with a PC level of 50% suffers from a peripheral arterial disease, while his son (IV-6) at the age of 30 developed DVT after minor trauma. The patient’s father (II-6) and his two brothers (II-2 and -3) died of MI, before the age of 50. In addition, renal artery aneurysms were diagnosed in the daughter of the proband’s father’s sister (III-1).

Among living members of this family, specifically in III-5 and III-2 members, a type I PC deficiency was diagnosed. The remaining thrombophilia testing showed normal antithrombin, PS, and FVIII, and the absence of antiphospholipid antibodies Factor V Leiden and *F2* gene g.20210 G>A mutations.

Interestingly, molecular analysis performed on the proband did not detect any known mutations associated with aneurysms, i.e., in genes encoding components of the extracellular matrix (*FBN1*, *COL3A1*), in loci encoding ligand receptors, and downstream effectors of the TGF-β signaling pathway (*TGFBR1*, *TGFBR2*, *SMAD3*, *SKI*), and in genes encoding proteins and enzymes involved in the contractile unit of vascular smooth muscle cells (*ACTA2*, *MYH11*) [14].

## 4. Discussion

In this case series, we characterize fourteen Polish patients with PC deficiency. Interestingly, in one proband, herein we report the identification of the novel c.401-1G>A mutation, which is located in intron 5, just upstream of exon 6, exactly within the acceptor splicing site. By destroying this canonical splice site, the new mutation prevents normal splicing. This can lead to the formation of transcripts including introns, partial deletions, or fully skipped exons, which yields truncated or otherwise non-functional proteins [15]. In the patient carrying a c.401-1A allele (Figure 1, III-2), the first clinical manifestation was the hepatic artery aneurysm, and thrombophilia screening was conducted due to a positive family history of VTE and coexisting cerebral aneurysm despite a negative personal history of thromboembolic events.

It should be noted that in the presented family, a woman, namely the proband (III-2) carrying the 401-1A variant and her cousin (III-1), had aneurysms in atypical locations. However, although several studies seem to suggest such a possibility, it is still unclear whether there is any association between *PROC* mutations and the presence of artery aneurysms at any location. Andreau et al. [16] indicated that aneurysms may be associated with other thrombophilia-predisposing mutations. In a study of 186 Greeks, they showed that certain thrombophilia-related mutations such as factor V Leiden and prothrombin c.20210G>A may contribute to the pathogenesis of intracranial aneurysms in a subset of the general population. Ozge et al. [17] reported a case of a 39-year-old patient with Behcet’s syndrome with the coexistence of PC and PS deficiency and bilateral pulmonary aneurysms. The patient neither had an APC resistance nor had the factor V Leiden mutation or the c.20210G>A prothrombin mutation. Navarro et al. [18,19] evaluated the PC system in subjects with Behçet’s disease and demonstrated that such patients who develop a VTE had significantly lower levels of plasma APC, PC inhibitor, and thrombomodulin than those patients who did not. In fact, APC levels below 0.75 ng/mL (10th percentile of the control group) increased the risk of VTE about fivefold (odds ratio = 5.1; 95% confidence interval 1.1–23.4) [18]. In addition, they evidenced that haplotypes of the endothelial PC receptor may modulate the thrombotic risk in Behçet’s disease patients [19]. These studies demonstrate the association between the protein C system and the thrombotic events in Behçet disease. Unfortunately, they did not evaluate the presence of aneurysms in these patients. Accordingly, the association of Behcet’s disease with PC and PS deficiencies might be further explored. In turn, Canpolat et al. [20] described the case of a 26-year-old patient with ascending aortic aneurysm diagnosed with Marfan syndrome, who experienced PE due to some genetic predisposition to thrombophilia. While adverse cardiovascular events are known in patients with Marfan syndrome, thromboembolic events are rarely reported. Furthermore, our proband with the new splice site *PROC* mutation did not have any known mutations predisposing to aneurysms. Simultaneously, further research indicates new evidence of a correlation between vascular anomalies and severe thrombophilia. De la Morena-Barrio et al. [21] showed the association of an antithrombin deficiency with atresia of inferior vena cava (IVC). Sixteen out of twenty four (66.7%) patients with antithrombin deficiency carrying the homozygous *SERPINC1* p.Leu131Phe mutation (antithrombin Budapest 3) had IVC system atresia, possibly caused by thrombosis in the developing fetal vessels. This evidence reinforces our finding highlighting a potential correlation between the *PROC* c.401-1G>A mutation and the presence of aneurysms.

Among the remaining thirteen patients studied herein, we detected eight mutations that had been previously found to be functionally associated with PC deficiency, including five mutations that were described previously in the Polish Slavic population, namely p.His253Gln, p.IVS5+2T>C, p.Arg348 *, p.Cys106Arg and p.Arg211Gln [9,22]. Moreover, like in the previous study [9], in the current case series, the p.IVS5+2T>C variant occurred in a single patient with an ischemic stroke. Isolated DVT or DVT with concomitant PE was the most common clinical manifestation of PC deficiency related to the mutations reported previously, including p.Arg348 *, p.Cys106Arg, p.Arg211Gln, and p.His253Gln. The last variant has also been found in a patient with cerebral venous sinus thrombosis [9].

One of the known mutations, p.Pro321Leu in exon 9, was described by Martos et al. [12] and indicated as potentially less prothrombotic in terms of age of the first thrombotic event. However, in our study, it was found in a proband who experienced VTE at a relatively young age (25 years) compared to the entire group (41.3 ± 12.1 years). Additionally, in our group of probands we recorded the p.Arg211Gln mutation in exon 7, located near the p.Pro210Arg and p.Pro210Leu, a second variant in the same residue, all causing a type I PC deficiency. As indicated previously, a mutation in Pro210 residues probably destabilizes the tertiary structure of the activation peptide [12]. Herein, we only found one mutation common to the probands from our study and Spanish patients with mutations of the *PROC* gene, which confirms the wide spectrum of these mutations in European countries [12].

During the follow-up period, we did not observe any thromboembolic event, which is consistent with findings of a series of 33 patients treated with NOACs after VTE associated with severe inherited thrombophilia [23]. Thus, our study supports that NOACs can be safe and effective for VTE treatment and (secondary) prevention in patients with inherited thrombophilias, including PC deficiency.

The study has several limitations. It should be highlighted that asymptomatic thromboembolic events may have gone unnoticed. Furthermore, in some cases, the levels of PC did not correlate with *PROC* abnormalities associated with deficiency, which is consistent with previous studies and indicates that genetic counseling is needed for these patients [12]. In a study on an association between *PROC* genotype and plasma or clinical phenotypes, Alhenc-Gelas et al. [24] showed that PC anticoagulant activity significantly influenced the risk of first VTE event. However, they found no relationship between the type of a mutation and the risk of unprovoked thrombotic events. Besides, we were not able to analyze all important members of the proband’s family.

In conclusion, fourteen Polish patients with PC deficiency were genetically characterized and a single new detrimental *PROC* mutation was identified in a family with aneurysms, which might suggest yet unclear links of thrombophilia to vascular anomalies, including aneurysms at atypical locations in women. The present case series supports data indicating that NOACs are effective in PC-deficient patients. Genetic evaluation of patients suspected of PC deficiency should be performed.

## Figures and Tables

**Table 1:** Characteristics of patients with protein C (PC) deficiency. Mutations were given coordinates according to the HUGO recommendations for mutation nomenclature (http://www.hgvs.org, accessed on 1 March 2022). Abbreviations: DVT denotes deep vein thrombosis; F, female; M, male; N/A, not applicable; N/D, not definable; PE, Pulmonary embolism; SVT, superficial vein thrombosis; VTE, venous thromboembolism. *: this is the marking of the terminal mutation.

Patient ID	Sex/Age	PC Activity % (Chromogenic Assay)	PC Activity % (Clot-Based Assay)	Total PC %	Type of PC Deficiency	Type of Mutation in *PROC* Gene	Exon Number	New/Reported	Clinical Manifestation	Age of First Thromboembolic Event	Number of VTE Events	Unprovoked/Provoked	Family History of VTE	Duration (Months)	Thrombo Embolic Events	Antithrombotic Treatment
1	F/56	45	34	N/D	I	c.401-1G>A	Intron 5	New	Hepatic artery aneurysm	51	N/A	N/A	1	54	0	ASA (75 mg/day)
2	M/54	65	56	118	II	c.759C>A, p.His253Gln	Exon 8	Reported	DVT	50	1	0/long journey	0	57	0	Dabigatran (150 mg bid)
3	F/44	56	N/D	N/D	II	c.759C>A, p.His253Gln	Exon 8	Reported	DVT	37	1	1/0	0	84	0	Sulodexid (2 × 250 SLU)
4	F/35	68	64	139	IIa	c.759C>A, p.His253Gln	Exon 8	Reported	DVT-cesarean section complicated with hemorrhage	33	1	0/pregnancy	1	7	0	Dabigatran (150 mg bid)
5	M/52	68	59	54	II	c.759C>A, p.His253Gln	Exon 8	Reported	DVT	47	2	1/0	0	18	0	Rivaroxaban (15 mg/day)
6	M/63	58	45	113	II	c.759C>A; p.His253Gln	Exon 8	Reported	DVT+PE	62	1	0/long journey	1	5	0	Rivaroxaban (20 mg/day)
7	F/64	67	57	46	I	c.400+2T>C	Intron 5	Reported	Ischemic stroke	59	0	1/0	1	54	0	Clopidogrel (75 mg/day)
8	F/39	72	50	N/D	N/A	c.1042C>T, p.Arg348 *	Exon 9	Reported	Asymptomatic	N/A	N/A	N/A	1	36	0	none
9	M/22	47	39	53	I	c.316T>C, p.Cys106Arg	Exon 5	Reported	DVT	20	1	1/0	0	13	0	Dabigatran (150 mg bid)
10	F/48	46	34	35	I	c.316T>C, p.Cys106Arg	Exon 5	Reported	DVT+PE	45	1	0/surgery	0	31	0	Apixaban (2.5 mg bid)
11	F/32	59	37	103	II	c.595C>T, p.Arg199 *	Exon 7	Reported	DVT+PE	30	1	0/oral contraceptives	0	26	0	Rivaroxaban (20 mg/day)
12	F/26	52; 56	50	54	I	c.962C>T, p.Pro321Leu	Exon 9	Reported	VTE	25	1	0/oral contraceptives	1	12	0	Apixaban (5 mg bid)
13	F/43	68	52	66.9	I	c.1174G>A p.Gly392Arg	Exon 9	Reported	PE	42	1	0/oral contraceptives	1	6	0	Apixaban (2.5 mg bid)
14	M/37	56	46	69.8	I	c.632G>A p.Arg211Gln	Exon 7	Reported	SVT	36	1	1/0	0	6	0	Rivaroxaban (20 mg/day)

## Data Availability

The data presented in this work are available on request from the corresponding author.
